# Comorbidity and dementia: A nationwide survey in Taiwan

**DOI:** 10.1371/journal.pone.0175475

**Published:** 2017-04-12

**Authors:** Ting-Bin Chen, Szu-Yu Yiao, Yu Sun, Huey-Jane Lee, Shu-Chien Yang, Ming-Jang Chiu, Ta-Fu Chen, Ker-Neng Lin, Li-Yu Tang, Chung-Chih Lin, Pei-Ning Wang

**Affiliations:** 1 Department of Neurology, Neurological Institute, Taichung Veterans General Hospital, Taichung, Taiwan; 2 Department of Biostatistics, Columbia University Mailman School of Public Health, New York, NY, United States of America; 3 Department of Neurology, National Taiwan University Hospital, Taipei, Taiwan; 4 Department of Neurology, En Chu Kong Hospital, New Taipei City, Taiwan; 5 Taiwan Alzheimer’s Disease Association, Taipei, Taiwan; 6 Graduate Institute of Brain and Mind Sciences, College of Medicine, National Taiwan University, Taipei, Taiwan; 7 Graduate Institute of Psychology, College of Science, National Taiwan University, Taipei, Taiwan; 8 Department of Psychology, Soo-Chow University, Taipei, Taiwan; 9 Department of Neurology, Neurological Institute, Taipei Veterans General Hospital, Taipei, Taiwan; 10 Department of Computer Science and Information Engineering, Chung Gung University, Tao-Yuan, Taiwan; 11 Department of Neurology, National Yang-Ming University School of Medicine, Taipei, Taiwan; 12 Aging and Health Research Center, National Yang-Ming University, Taipei, Taiwan; Banner Alzheimer's Institute, UNITED STATES

## Abstract

**Background:**

Comorbid medical diseases are highly prevalent in the geriatric population, imposing hardship on healthcare services for demented individuals. Dementia also complicates clinical care for other co-existing medical conditions. This study investigated the comorbidities associated with dementia in the elderly population aged 65 years and over in Taiwan.

**Methods:**

We conducted a nationwide, population-based, cross-sectional survey; participants were selected by computerized random sampling from all 19 Taiwan counties between December 2011 and March 2013. After exclusion of incomplete or erroneous data, 8,456 subjects were enrolled. Of them, 6,183 were cognitively normal (control group), 1,576 had mild cognitive impairment (MCI), and 697 had dementia. We collected information about types of comorbidities (i.e., vascular risk factors, lung diseases, liver diseases, gastrointestinal diseases, and cancers), Charlson comorbidity index score, and demographic variables to compare subjects with normal cognition, MCI, and dementia.

**Results:**

Regardless of the cognitive condition, over 60% of the individuals in each group had at least one comorbid disease. The proportion of subjects possessing at least three comorbidities was higher in those with cognitive impairment (MCI 20.9%, dementia 27.3%) than in control group (15%). Hypertension and diabetes mellitus were the most common comorbidities. The mean number of comorbidities and Charlson comorbidity index score were greater in MCI and dementia groups than in control group. Logistic regression demonstrated that the comorbidities significantly associated with MCI and dementia were cerebrovascular disease (OR 3.35, CI 2.62–4.28), cirrhosis (OR 3.29, CI 1.29–8.41), asthma (OR 1.56, CI 1.07–2.27), and diabetes mellitus (OR 1.24, CI 1.07–1.44).

**Conclusion:**

Multiple medical comorbid diseases are common in older adults, especially in those with cognitive impairment. Cerebrovascular disease, cirrhosis, asthma, and diabetes mellitus are important contributors to cognitive deterioration in the elderly. Efforts to lower cumulative medical burden in the geriatric population may benefit cognitive function.

## Introduction

The incidence of dementia increases with age and puts a profound socioeconomic burden on care-givers and healthcare systems in Taiwan [1,2]. A nationwide, population-based, cross-sectional survey of the Taiwanese population aged 65 years or older between 2011 and 2013 demonstrated that the age-adjusted prevalence of mild cognitive impairment (MCI) was 18.7%, and that of all-cause dementia was 8.04%; its frequency increases strongly with age, from 3.4% in people aged 65–69 years to 36.8% in those aged 90 years or older [3]. With the population aging, the number of people with dementia in Taiwan is estimated to increase substantially, doubling every 20 years, and is expected to reach 0.32 million by 2030 and to exceed 0.6 million by 2050 [4].

Although dementia cannot be cured and ultimately may lead to severe disability and institutionalization, reducing the risk of developing dementia takes on added importance in the absence of a disease-modifying treatment. Previous studies have highlighted that a number of non-modifiable risk factors, such as age and apolipoprotein E genotype, were strongly associated with dementia [5]. Emphasis should be placed on understanding which potentially modifiable lifestyle and medical factors might alter the risk of dementia. Identifying these risk factors that hasten the onset and progression of dementia is crucial for timely medical intervention and predicting prognosis. Improved knowledge of the comorbidities of highly prevalent chronic health problems in patients with dementia would facilitate the development of preventive strategies aimed at slowing or avoiding rapid clinical and functional deterioration. Therefore, risk factor modification and reduction will become a fundamental strategy in clinical practice and a focus of public health efforts, in view of the increasing longevity of populations worldwide.

Patients with dementia have, on average, 2 to 8 additional chronic comorbid illnesses [6,7]. Comorbidities not only complicate dementia care and decrease health-related quality of life, but also might reveal shared mechanisms between disorders and provide insight into dementia pathogenesis. Accumulating evidence has shown that vascular risk factors (VRFs), including hypertension, diabetes mellitus (DM), hyperlipidemia, and cerebrovascular disease, are associated with cognitive impairment and dementia [8–11]. Aside from VRFs, several chronic medical disorders, such as hypertension, chronic heart failure, arrhythmia, and diabetes mellitus, have been reported to be related to cognitive dysfunction and brain pathology and even to co-occur in dementia, interacting with each other [12,13].

Similar to Western societies, age, sex, family history of dementia, and apolipoprotein E epsilon 4 allele are proven to be the major risk factors for dementia in Taiwan [14–17]. However, none of these risk factors can be modified by medical interventions or by individual behavior. Until now, there have not been any reports regarding the relationship between concurrent chronic disorders and developing dementia in the Taiwanese population. To address this issue and pursue preventive approaches, we conducted the first nationwide, population-based, cross-sectional survey in Taiwan to investigate the epidemiology and risk factors in dementia and the association of comorbid illnesses with mild cognitive impairment (MCI) and dementia among people aged 65 years and older.

## Methods

### Study design and sampling

This study was a nationwide, population-based, cross-sectional survey with participants enrolled between December 2011 and March 2013. With the assistance of the Ministry of Health and Welfare of Taiwan and local city governments, our study team obtained the address lists needed. All participants aged 65 years and older were randomly sampled and recruited to achieve a nationally representative sample. The details of this study are described elsewhere [3,18]. This study was approved by the ethics committee at the National Taiwan University Hospital. Permission for interview and written informed consent were obtained from each elder but if that was not possible, from the closest responsible adult. In addition, verbal consent was asked from the participant and the informant when the study was explained verbally at the start.

All the subjects completed the survey using door-to-door screenings [3]. We performed in-person interviews to collect a brief history focusing on cognitive and functional status, followed by a structured questionnaire recording demographic data, medical comorbidities, lifestyle habit factors, and mental tests. The medical comorbid conditions were categorized as vascular risk factors (i.e., hypertension, DM, cerebrovascular disease, and hyperlipidemia), lung diseases, liver diseases, gastrointestinal diseases, and cancers. The lifestyle habit factors, including social activity, regular exercise, smoking, drinking, chewing betel nuts, sleep habits, and marital status, were recorded based on the definitions [18]. The interview process was performed according to an operational manual that defines all variables examined in this questionnaire. Logic checks for inconsistency and auditing were performed by experienced supervisors to ensure the quality and reliability of the entered data [3]. A medical history was taken to detect any insidious change of behavior or personality and any mental decline from previous levels of functioning [3].

### Diagnostic criteria

The diagnosis for all-cause dementia was based on the core clinical criteria recommended by the National Institute on Aging-Alzheimer’s Association (NIA-AA). Cognitive and functional status were determined from the evaluation with the participant or a knowledgeable informant who had taken care of the people with dementia for more than 10 hours a week and were capable of detecting insidious changes in behavior, personality, decline in mentality or function at work, or activities of daily living (ADL). Objective assessments included the Clinical Dementia Rating Scale (CDR) and the Taiwanese Mini-Mental State Evaluation (T-MMSE). Normal T-MMSE results were defined as a score >24 in literate elderly people and >13 in illiterate elderly people [19]. Participants who were compatible with the NIA-AA criteria for all-cause dementia were those with impairment in 2 or more cognitive domains as well as a decline in daily functions, whereby the cognitive deficits were sufficient to interfere with independence of daily living function as a result of abnormality in community affairs or at-home hobbies or personal care, as assessed by the CDR. Functional status was assessed using the ADL scale and the instrumental activities of daily living (IADL) scale. MCI was diagnosed, based on the criteria recommended by the NIA-AA, as a change in cognition with impairment in 1 or more cognitive domains but no evidence of impairment in social or occupational functioning as assessed by the CDR, ADL, and IADL [20]. An individual was considered cognitively normal when one had none of the conditions listed in the NIA-AA core clinical criteria for all-cause dementia and have a CDR score of 0 as well as an education adjusted T-MMSE within normal limits. People with major depression, other mental disorders, delirium, or other serious physical problems leading to cognitive or functional status impairment did not fulfill the NIA-AA criteria for all-cause dementia and MCI and were therefore excluded.

The diagnosis of dementia, particularly difficult cases, was re-examined and discussed by a consultant panel consisting of four neurologists and one clinical psychologist specialized in the diagnosis and management of patients with dementia. The sampling process, training of interviewers, home visiting procedure, quality control, and protocol approvals were detailed in our previous report [3]. All interviews were conducted by well-trained field interviewers with continuous quality control to achieve necessary quality standards. The inter-rater reliability of global Clinical Dementia Rating was substantial, with a kappa value of 0.671 [3].

### Statistical analysis

Continuous variables are reported as means ± standard deviations. Categorical variables are represented by frequency or percentage. The Charlson comorbidity index (CCI) and number of comorbidities were analyzed to determine overall systemic health [21]. In the CCI, dementia was not taken into account given its presence in the cohort study. Univariate logistic regression analysis was used to assess the association between all comorbidity variables and MCI or dementia and to calculate crude odds ratio (OR) and its 95% confidence interval (CI). Factors with a *p*-value < 0.1 in univariate regression analyses were entered into the multivariate analyses. Multivariate logistic regression model 1 was adjusted for age group, sex, education level, and body mass index category. Model 2 represented model 1 plus adjustment for lifestyle habit factors. We calculated the adjusted OR value and its 95% CI for MCI and dementia. All analyses were performed using SAS statistical software (version 9.4) with 2-tailed statistical tests.

## Results

Of the 10,571 subjects interviewed, 2,118 were excluded due to incomplete or possibly erroneous data, and finally, 8,456 subjects were enrolled. Of the 8,456 participants, 697 (8.2%) fulfilled the NIA-AA core clinical criteria for all-cause dementia and 1,576 (18.6%) fitted the criteria for MCI, whereas 6,183 (73.1%) were cognitively normal. Subjects with MCI and dementia were approximately three years and seven years older than those with normal cognitive function, respectively. Table 1 shows the demographic characteristics of all participants.

**Table 1:** Demographic data of study participants (n = 8,456).

			Normal (n = 6,183)		MCI (n = 1,576)		Dementia (n = 697)
**Continuous variables, n, %**		Mean	SD	Mean	SD	Mean	SD
Age		74.85	6.04	77.55	6.70	81.61	7.63
	Men	75.42	6.24	78.30	6.86	81.27	7.74
	Women	74.25	5.76	77.06	6.55	81.80	7.56
**Categorical variables, n, %**		n	%	n	%	n	%
Women		3,007	48.63	951	60.34	443	63.56
Age, years							
	65–74	3,339	54.00	571	36.23	134	19.23
	75–84	2,397	38.77	747	47.40	304	43.62
	≥ 85	447	7.23	258	16.37	259	37.16
Education, years							
	0	2,078	33.61	928	58.88	417	59.83
	1–6	2,391	38.67	439	27.86	180	25.82
	7–12	1,201	19.42	149	9.45	70	10.04
	> 12	513	8.30	60	3.81	30	4.30
Body Mass Index (BMI)^a^							
	≤ 18	140	2.26	48	3.05	23	3.30
	18< BMI ≤ 24	2,853	46.14	591	37.50	311	44.62
	24< BMI ≤ 30	2,299	37.18	525	33.31	178	25.54
	> 30	258	4.17	64	4.06	27	3.87

MCI: mild cognitive impairment; SD, standard deviation.

^a^ There were 1,139 subjects with missing data in the BMI category.

Table 2 represents the detailed information of each comorbid illness among the three study groups. Overall, hypertension and DM were the most common comorbid diseases. The mean number of comorbidities in the population with MCI (1.51 ± 1.37) and dementia (1.73 ± 1.50) was significantly higher than that in the population with normal cognition (1.29 ± 1.24). Among the elderly with normal cognition, 68.3% had one or more comorbidities and 15.6% possessed at least three. Among the subjects with MCI, 72.4% had at least one comorbid diseases and 20.9% possessed at least three. Among the participants with dementia, 75.5% had one or more comorbid diseases and 27.3% possessed at least three.

**Table 2:** Comorbidities of study participants (n = 8,456).

			Normal (n = 6,183)		MCI (n = 1,576)		Dementia (n = 697)	
Categorical variables, n, %			n	%	n	%	n	%
Comorbidities								
	Vascular risk factor							
		Hypertension	3,075	49.70	833	52.86	369	52.94
		Diabetes mellitus	1,204	19.47	356	22.59	214	30.70
		CVD	204	3.30	122	7.74	129	18.51
		Hyperlipidemia	1,157	18.71	342	21.70	117	16.79
	Lung disease		282	4.56	100	6.35	77	11.05
		Tuberculosis	43	0.70	9	0.57	12	1.72
		Pneumonia	22	0.36	9	0.57	12	1.72
		COPD	16	0.26	7	0.44	6	0.86
		Asthma	123	1.99	48	3.05	32	4.59
		Benign lung tumor	20	0.32	5	0.32	3	0.43
		Other lung diseases	56	0.91	21	1.33	17	2.44
	Liver disease		284	4.59	90	5.71	33	4.73
		Hepatitis	164	2.65	51	3.24	16	2.30
		Cirrhosis	12	0.19	8	0.51	5	0.72
		Benign liver tumor	19	0.31	5	0.32	2	0.29
		Diagnosed liver disease	73	1.18	21	1.33	10	1.43
	Gastrointestinal disease		1,008	16.30	300	19.04	110	15.78
	Cancer		280	4.53	59	3.74	53	7.60
		Liver cancer	15	0.24	6	0.38	4	0.57
		Cervical cancer	20	0.32	6	0.38	2	0.29
		Colorectal cancer	55	0.89	10	0.63	11	1.58
		Breast cancer	47	0.76	8	0.51	8	1.15
		Lung cancer	17	0.27	3	0.19	7	1.00
		Gastric cancer	17	0.27	6	0.38	3	0.43
		Other cancer	112	1.81	24	1.52	17	2.44
	Number of comorbidities^a^							
		0	1,961	31.7	436	27.6	171	24.5
		1	1,938	31.3	451	28.6	184	26.4
		2	1,314	21.2	359	22.7	152	21.8
		≥ 3	970	15.6	330	20.9	190	27.2
		Mean number ± SD^b^	1.29 ± 1.24		1.51 ± 1.37		1.73 ± 1.50	
	CCI^a^							
		Score 0	4,471	72.31	1043	66.18	357	51.22
		Score 1	1,185	19.17	364	23.10	225	32.28
		Score 2	382	6.18	123	7.80	82	11.76
		Score 3	94	1.52	29	1.84	19	2.73
		Score 4	39	0.63	7	0.44	6	0.86
		Score ≥ 5	12	0.19	10	0.63	8	1.15
		Mean score ± SD^b^	0.40 ± 0.75		0.49 ± 0.83		0.73 ± 0.96	

MCI, mild cognitive impairment; CVD, cerebrovascular disease; COPD, chronic obstructive pulmonary disease; SD, standard deviation; CCI, Charlson comorbidity index.

^a^ *p* < .0001 by Chi-square test to compare group differences.

^b^ *p* < .0001 for normal vs. MCI, normal vs. dementia, MCI vs. dementia by ANOVA with Tukey’s post hoc test.

The severity of comorbid diseases was scored according to CCI. The mean CCI score in the participants with MCI (0.49 ± 0.83) and dementia (0.73 ± 0.96) was significantly greater than that in the cognitively normal subjects (0.40 ± 0.75). The distribution patterns of the number of comorbidities and CCI score were different across three study groups (Table 2). The proportion of those with index scores of 1–2, 3–4, and ≥ 5 was significantly higher in those with MCI and dementia than in the normal control group (Table 2).

Table 3 demonstrates the association between each comorbid illness with MCI and dementia using univariate logistic regression analyses. Associated factors with increased odds for both MCI and dementia were DM, cerebrovascular disease, lung disease, asthma, and cirrhosis. Factors only associated with MCI were hypertension, hyperlipidemia, and gastrointestinal disease, and only with dementia included tuberculosis, pneumonia, chronic obstructive pulmonary disease, other lung disease, and cancer.

**Table 3:** Crude ORs of all comorbidities for MCI and dementia using univariate logistic regression analyses.

Variables		MCI vs. Normal			Dementia vs. Normal		
		Crude ORs	*p* value	95% CI	Crude ORs	*p* value	95% CI
Vascular risk factor							
	Hypertension	1.13	0.03	1.01–1.27	1.14	0.11	0.97–1.33
	Diabetes mellitus	1.21	0.01	1.06–1.38	1.83	< .01	1.52–2.18
	Cerebrovascular disease	2.46	< .01	1.95–3.10	6.66	< .01	5.26–8.44
	Hyperlipidemia	1.20	0.01	1.05–1.38	0.88	0.21	0.71–1.08
Lung disease		1.42	< .01	1.12–1.79	2.60	< .01	1.99–3.39
	Tuberculosis	0.82	0.59	0.40–1.69	2.50	< .01	1.31–4.77
	Pneumonia	1.61	0.23	0.74–3.50	4.91	< .01	2.42–9.96
	COPD	1.72	0.23	0.71–4.19	3.35	0.01	1.31–8.59
	Asthma	1.55	0.01	1.10–2.17	2.37	< .01	1.59–3.53
	Benign lung tumor	0.98	0.97	0.37–2.62	1.33	0.64	0.40–4.50
	Other lung diseases	1.48	0.13	0.89–2.45	2.74	< .01	1.58–4.74
Liver disease		1.26	0.06	0.99–1.61	1.03	0.87	0.71–1.49
	Hepatitis	1.23	0.21	0.89–1.69	0.86	0.58	0.51–1.45
	Cirrhosis	2.62	0.03	1.07–6.43	3.72	0.01	1.31–10.58
	Benign liver tumor	1.03	0.95	0.39–2.77	0.94	0.93	0.22–4.02
	Diagnosed liver disease	1.13	0.62	0.69–1.84	1.22	0.56	0.63–2.37
Gastrointestinal disease		1.21	0.01	1.05–1.39	0.96	0.72	0.78–1.19
Cancer		0.82	0.17	0.62–1.09	1.74	< .01	1.28–2.35
	Liver cancer	1.57	0.35	0.61–4.06	2.37	0.13	0.79–7.17
	Cervical cancer	1.18	0.73	0.47–2.94	0.89	0.87	0.21–3.80
	Colorectal cancer	0.71	0.32	0.36–1.40	1.79	0.08	0.93–3.43
	Breast cancer	0.67	0.29	0.31–1.41	1.52	0.28	0.71–3.22
	Lung cancer	0.69	0.56	0.20–2.37	3.69	< .01	1.52–8.92
	Gastric cancer	1.39	0.49	0.55–3.52	1.57	0.47	0.46–5.37
	Other cancer	0.84	0.44	0.54–1.31	1.36	0.25	0.81–2.27

MCI, mild cognitive impairment; COPD, chronic obstructive pulmonary disease; CI, confidence interval; ORs, odds ratios.

After adjusting for sex, age, educational level, BMI (model 1, Table 4) or additional lifestyle habit factors (model 2, Table 4), cerebrovascular disease, cirrhosis, asthma, and DM were still associated with MCI and dementia. These four risk factors remained consistent with model 1 and model 2 when grouping both MCI and dementia as cognitive impairment.

**Table 4:** Adjusted ORs for association with cognitive impairment regarding comorbidities.

		Model 1		Model 2	
Variables		ORs (95%CI)	*p* value	ORs (95%CI)	*p* value
Comorbidity					
	Hypertension	0.99 (0.88–1.12)	0.88	0.98 (0.86–1.11)	0.77
	Diabetes mellitus	1.27 (1.10–1.46)	< .01	1.24 (1.07–1.44)	< .01
	Cerebrovascular disease	3.66 (2.89–4.64)	< .01	3.35 (2.62–4.28)	< .01
	Hyperlipidemia	0.99 (0.85–1.16)	0.92	0.98 (0.83–1.15)	0.81
	Tuberculosis	1.37 (0.74–2.54)	0.31	1.58 (0.84–2.97)	0.16
	Pneumonia	1.96 (0.94–4.07)	0.07	1.94 (0.91–4.12)	0.09
	Chronic obstructive pulmonary disease	2.17 (0.86–5.43)	0.10	1.88 (0.73–4.87)	0.19
	Asthma	1.65 (1.15–2.38)	< .01	1.56 (1.07–2.27)	0.02
	Other lung diseases	1.58 (0.94–2.65)	0.08	1.54 (0.90–2.63)	0.12
	Cirrhosis	4.04 (1.62–10.08)	< .01	3.29 (1.29–8.41)	0.01
	Gastrointestinal disease	1.00 (0.86–1.18)	0.96	1.01 (0.86–1.19)	0.91
	Cancer	1.10 (0.80–1.52)	0.56	0.99 (0.71–1.39)	0.96
	Colorectal cancer	0.79 (0.40–1.59)	0.51	0.86 (0.42–1.78)	0.69
	Lung cancer	1.40 (0.48–4.11)	0.54	1.36 (0.43–4.27)	0.60

Model 1 indicates model adjusted for age group, gender, education level, and BMI category.

Model 2 indicates model adjusted for age, gender, education level, BMI category, and lifestyle habit factors.

CI: confidence interval; ORs: odds ratios.

Table 4 illustrates the adjusted ORs for association with cognitive impairment regarding comorbidities. In comparison with people without cerebrovascular disease, the adjusted OR of cognitive impairment was 3.35 (95% CI = 2.62–4.28) in those with cerebrovascular disease. Compared with people without cirrhosis, the OR in those with cirrhosis was 3.29 (95% CI = 1.29–8.41). The OR was 1.56 (95% CI = 1.07–2.27) in subjects with asthma, compared with those without asthma. Compared with people without DM, the OR in people with DM was 1.24 (95% CI = 1.07–1.44).

## Discussion

In this population-based, cross-sectional survey, over 60% of both cognitively preserved and cognitively impaired older individuals had comorbid medical conditions. Hypertension and DM were the most frequent comorbid diseases. The magnitude and pattern of comorbidity strongly involve cognitive impairment, particularly MCI and dementia. We also report evidence that cerebrovascular disease, cirrhosis, asthma, and DM increase the odds for cognitive impairment.

Higher medical comorbidity not only accelerates functional deterioration leading to the under-diagnosis and under-treatment of dementia and comorbid illnesses, but also has significant implications for poorer self-care, immobility, and polypharmacy [22–24]. Our study showed the burden of comorbidities is significantly high in older adults with cognitive impairment.

Several studies concerning comorbidities in subjects with cognitive impairment were conducted in Japan [25], Korea [26], the United States [6,7], the United Kingdom [27], France [28], and Spain [22,29]. The cross-national comparison of detailed information regarding the comorbidity and CCI score is presented in Table 5. Taken as a whole, across different countries, the mean CCI score is 2, but the number and item of common comorbidities vary. Similar to studies from Japan, Korea, and France, we showed that hypertension, DM, and hyperlipidemia are the most common comorbidities among subjects with cognitive impairment [25,26,28].

**Table 5:** Cross-national comparison of comorbidities in cognitive impairment.

	n	Female,%	Age, mean	CCI, mean ± SD	number of comorbidity	Common comorbidities
Taiwan	2,273	61.3	MCI: 77.5 Dementia: 81.6	MCI: 0.4 Dementia: 0.7^a^	MCI: 1.51 Dementia: 1.73	Cerebrovascular disease, Cirrhosis, Asthma, DM
Japan (2010)	113	76.1	78.6	–	2.27	Hyperlipidemia, Hypertension
Korea (2011)	1,786	67	71.4	–	–	Hypertension, Anemia, DM
US (2002)	15,013	69.8	–	–	7.2–8.13	Femoral neck fracture, Urinary tract infection, Convulsion
US (2006)	107	62.6	75.6	–	2.4	Hypertension, DM
UK (2011)	1,486	52.1	MCI: 73 Dementia: 80	–	–	
France (2005)	579	72	77.4	1.5 ± 0.9	–	Hypertension, Hyperlipidemia
Spain (2009)	515	70	81	2 ± 1.2	–	
Spain (2014)	3,971	70.1	80.2	–	3.69	Anxiety, Chronic skin ulcers, Parkinson’s disease, Anemia, Cerebrovascular disease, Cardiac arrhythmia, Thyroid disease

CCI, Charlson comorbidity index; DM, diabetes mellitus;–, not available.

^a^ dementia is not taken into account, given its presence in dementia group.

A large body of literature has indicated that several VRFs play common convergent roles in vascular and neurodegenerative cognitive impairment [30,31]. Our study showed cerebrovascular disease provides the highest risk of MCI and dementia. Cerebrovascular changes, such as hemorrhagic infarct, small and large cortical infarct, vasculopathy, and white matter change, considerably impede trophic coupling in neurovascular units and cause cognitive decline [32,33].

DM was the other VRF associated with increased risk of cognitive impairment in our study. Disturbance in insulin signaling is the underlying mechanism in DM, and it affects cell growth, cerebral energy homeostasis, blood-brain barrier integrity, glial function, oxidative stress, and inflammatory response in the central nervous system [34–36]. Through the common pathway of insulin resistance and complex metabolic disorders, DM is notably associated with cognitive decline in the elderly [37].

Cirrhosis was an independent risk factor for cognitive impairment in our study. A wide range of neurocognitive disorders in cirrhotic patients with or without overt hepatic encephalopathy may be caused by a direct influence of cirrhosis on the nervous system or by substances or diseases that act on the brain and liver concurrently (e.g., alcohol or Wilson’s disease) or by hepatitis C virus-induced irreversible neurodegenerative damage [38,39].

As for asthma, our result was in line with the finding that asthma significantly increased the risk of dementia in one Taiwan nationwide cohort study [40]. In another Taiwan nationwide longitudinal study, asthma in midlife and in late life increased the risk of cognitive impairment [41]. The impact of chronic poor pulmonary function on cognition may be the direct consequence of pulmonary limitation and overlapping risk factors in both general and chronic lung disease populations [42].

Unlike previous reports, hypertension and hyperlipidemia were not associated with cognitive decline in our cohort. The association of hypertension with the risk of dementia is significant in middle-aged populations (around 50 years old), rather than in the elderly population aged 65 years and older [43]. Blood pressure was shown to decrease several years before the onset of dementia [44]. It might support a nonlinear, age-dependent relationship between blood pressure and the risk of cognitive decline in the elderly. On the other hand, cholesterol level was shown to be conversely related to the risk of MCI and dementia in the elderly population aged 65 and older [45]. This finding may be due to a decrease in total cholesterol with age, insufficient nutrition, and decline in BMI several years before onset of dementia [46]. Moreover, in our cohort, most participants with hypertension or hyperlipidemia have been prescribed with antihypertensive or lipid-lowering agents, respectively. This may imply that medical control of hypertension and hyperlipidemia is beneficial for the risk of developing dementia.

There are some limitations in this study. First, there is the potential for diagnostic bias and misclassification of comorbidities because the medical history was taken from the non-demented participant or based on the medical record documentation or chronically-prescribed drugs, instead of a formal diagnostic assessment by clinicians. Second, the definition of comorbidity itself is complex. The impact of a given comorbidity on cognition varies in terms of the duration and severity of the comorbidity before the onset of dementia, pharmacotherapy, and synergistic effect of other comorbidities. Third, we did not group enrolled participants into different MCI subtypes and dementia subtypes. Different subtypes of cognitive impairment may be related to distinct risk factors, thus complicating the identification of direct relationships. Fourth, as the common limitation of all cross-sectional studies, the corroboration of the causality between comorbidity and cognitive impairment warrants further investigation in longitudinal studies.

## Conclusions

Multiple medical comorbid diseases are common in older adults with cognitive impairment. Identification and treatment of manageable comorbidities are important to delay the progression of the disability from dementia. Those with cerebrovascular disease, cirrhosis, asthma, and diabetes mellitus have higher likelihood of cognitive decline. Due to a constellation and complexity of comorbidities, greater attention is required to improve a comprehensive, tailored, and patient-centered approach for dementia care.
